# Investigation of the Effect of Mobile and Immobile Regions on Fat Graft Viability: An Experimental Study in a New Model

**DOI:** 10.1007/s00266-024-04267-9

**Published:** 2024-08-08

**Authors:** Gökçen Çakır Bozkurt, Levent Yoleri, Peyker Temiz

**Affiliations:** 1 Department of Plastic, Reconstructive and Aesthetic Surgery Manisa, Faculty of Medicine, Manisa Celal Bayar University, Manisa, Turkey; 2 Department of Pathology, Faculty of Medicine, Manisa Celal Bayar University, Manisa, Turkey

**Keywords:** Fat graft survival, Immobile, Mobile

## Abstract

**Background:**

Fat grafts are widely used in plastic, aesthetic and reconstructive surgery. Their unpredictable resorption is their main disadvantage. A review of the literature shows that there is a lack of research on the effect of mobile and immobile regions on fat graft survival in fat graft applications.

**Objective:**

Our aim was to investigate the relationship of fat graft survival with mobile and immobile region in a new experimental model.

**Methods:**

Twenty-four male Wistar albino rats were randomly divided into two groups (*n*=12). Fat grafts were harvested from the right inguinal region of the rat. In Group 1, the fat graft was placed in the subcutaneous pouch formed in the scalp region of the rat. In Group 2, fat grafts were placed in the pouch formed in the posterior cervical region of the rat. At the end of 6 weeks, the weights and histopathology of the fat grafts were evaluated. Histopathological examinations were performed in a blinded fashion.

**Results:**

The weights of the fat grafts were found to be higher in Group 1. At the same time, histopathological examinations showed that vascular density was higher in Group 1. There was no statistically significant difference in other histopathological examinations.

**Conclusion:**

The mobile and immobile areas may have different effects on the survival of transplanted fat grafts. Sliding movement between muscle and skin in the mobile zone puts stress on the fat graft. In our study, the mobile site was shown to have a negative effect on the vascularity of the fat graft. It was observed that the vascular density was higher in the fat graft placed in the immobilised area. Further studies on the increase in vascularity can be carried out using the new experimental model we have created.

**No Level Assigned:**

This journal requires that authors assign a level of evidence to each submission to which Evidence-Based Medicine rankings are applicable. This excludes Review Articles, Book Reviews, and manuscripts that concern Basic Science, Animal Studies, Cadaver Studies, and Experimental Studies. For a full description of these Evidence-Based Medicine ratings, please refer to the Table of Contents or the online Instructions to Authors www.springer.com/00266.

## Introduction

In the practice of plastic, aesthetic and reconstructive surgery, autologous fat grafts are often used for tissue augmentation, body contouring, facial rejuvenation and scar treatment. The advantages of autologous fat grafts are that they are easy to obtain, reproducible, easy to apply and does not cause a foreign body reaction [1]. The main disadvantages of autologous fat grafting are that it is not always possible to obtain sufficient volume, and there is unpredictable resorption at the site of the fat graft [2]. Studies suggest that the survival rate of fat grafts varies between 10% and 80% [3]. Therefore, many studies have been conducted in the literature to increase fat graft survival and decrease resorption rates [4]. The characteristics of the recipient site are also very important in fat graft applications. It has been observed that there are not enough studies in the literature to investigate the effect of recipient site mobility on fat graft viability [27].

One study investigated the effect of botulinum toxin A application on fat grafting. A fat graft was placed in the area where botulinum toxin A had been applied, and as a result of the examination, it was suggested that angiogenesis and adipogenesis were increased by decreasing muscle movement in the area where botulinum toxin A had been applied. Long-term survival of the fat graft in the supramuscular plane was reported to increase [5]. The author suggested that this was due to the immobilisation of the muscle. However, it may also be due to other effects of botulinum toxin A.

Another study performed temporary and permanent denervation of the latissimus dorsi muscle and fat grafting to these areas and investigated the effect of muscle immobilisation on fat grafting. This study reported that permanent damage to the thoracodorsal nerve resulted in muscle loss and severe loss of fat graft [6]. In this study, it was thought that fat graft loss could occur due to various effects of denervation [28].

In another study, immobilisation by denervation was performed on the hind legs of rats. It was observed that fat was injected into the muscle and subcutaneous tissue in the immobile and mobile regions. It was suggested that intramuscular fat injection increased ADSC density and new vessel formation in the immobile group. It was found that there was no significant difference between mobile and immobile regions in subcutaneous fat injection. The author suggested that the mechanical force and sliding movement affected the newly formed fragile vessels and therefore the vessels could not form and fat graft losses occurred due to malnutrition of the fat cells. The author stated that there are studies showing that mechanical force inhibits the proliferation and differentiation process of ADSCs [7]. The relationship between the experimental model and clinical practice is not appropriate. At the same time, we believe that the failure of neural pathways in the denervation method may lead to different effects [28].

Our study used a new experimental rat model to investigate the relationship between fat graft survival and whether the recipient site was mobile or immobile.

## Material and Methods

### Animal Model

The study was approved by the laboratory animal ethics committee. Twenty-four male Wistar albino rats weighing 300–350 g were used in the study. Fat grafts were harvested from the right inguinal region of each rat. There were two groups in the study. Each group consisted of 12 rats.

**Fig. 1:**
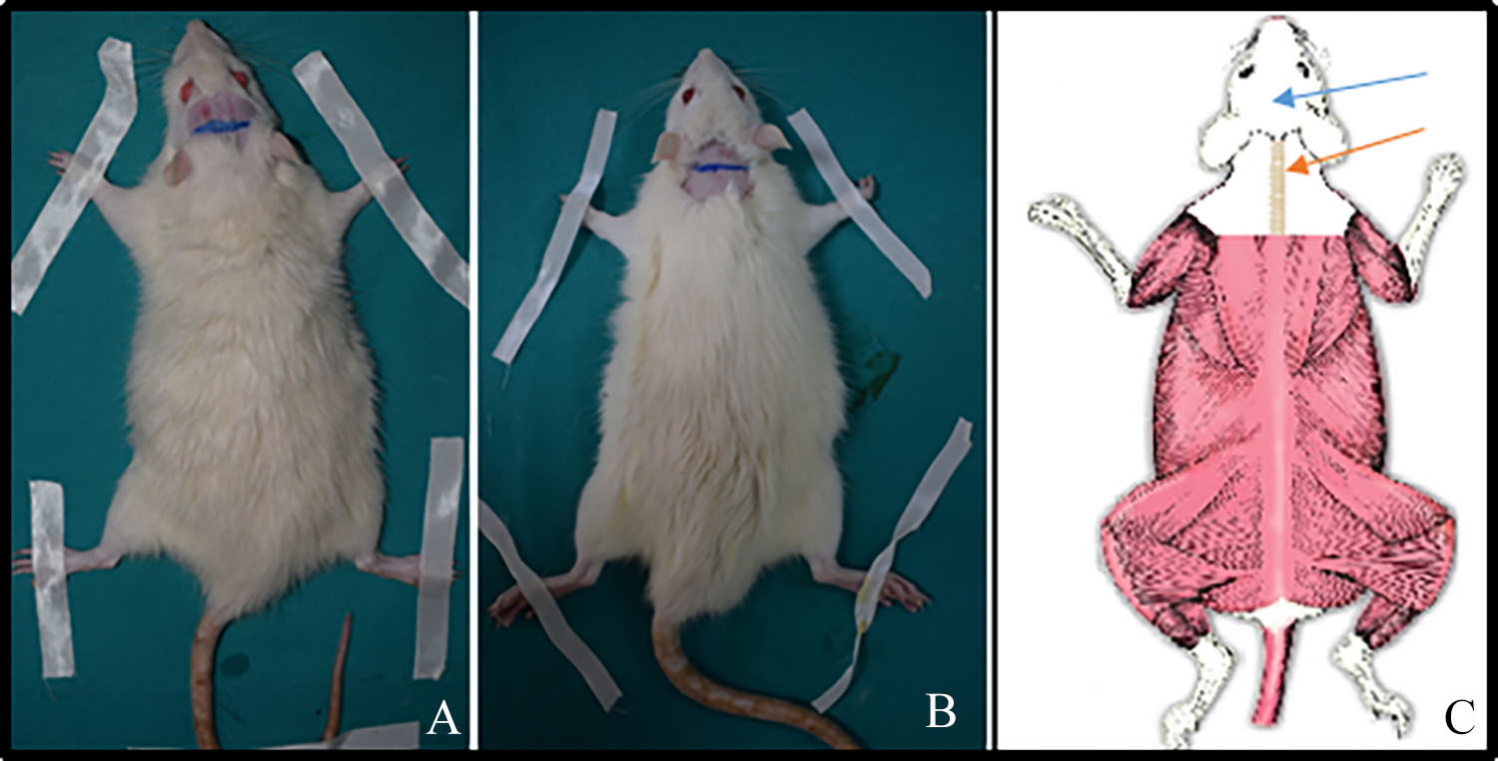
**A** Marking in the coronal plane, in front of both ears on the scalp as an immobile area of Group 1. **B** Marking in the coronal plane, in the cervical region as a mobile area of Group 2, in the posterior part of both ears. **C** The blue arrow on the drawing indicates the recipient site where the fat graft will be placed in Group 1 and the orange arrow indicates the recipient site where the fat graft will be placed in Group 2

In Group 1, the recipient site was the scalp region, which is considered to be the most immobile region of the rat. It was planned to open a pouch anterior to the incision line in the scalp region, in front of both ears. In Group 2, the recipient site was defined as the cervical dorsal region of the rat, which is mobile. The cervical dorsal region was chosen because it is adjacent to the scalp and its circulation is the same as the arterial structure supplying the scalp. In the cervical dorsal region, an incision was planned on the posterior side of both ears. A pouch was planned to be opened on the caudal side of the incision. A new experimental model was established to evaluate the survival of the fat graft (Fig. 1). Rats were housed in individual cages at standard room temperature and fed ad libitum.

### Surgical Methods

75 mg/kg ketamine and 10 mg/kg xylazine were injected intraperitoneally on the left side to achieve general anaesthesia. Cefazolin sodium 15/mg/kg was injected intramuscularly prophylactically. The hair of the right groin was shaved. Povidone-iodine was used to ensure sterility. After sterile dressing, a 3-cm skin incision was made in the right inguinal region, parallel to the inguinal ligament, and the inguinal fat pads were reached after dissection with scissors. The fat tissue was dissected from the surrounding tissues and harvested as a single piece. After bleeding control, the skin was repaired with 4/0 non-absorbable monofilament sutures. The donor area was dressed with povidone-iodine.

The harvested fat grafts were weighed on a precision balance and reduced to 0.5 g each [8].

*Group 1 (n*=12*):* Rats were restrained in the prone position. The scalp region anterior to both ears and posterior to both eyes, designated as the immobile region, was shaved. After cleaning the area with povidone-iodine, a 1.5-cm coronal incision was made in the scalp skin. A 1.5 × 1 cm bag was opened anteriorly directly on the periosteum in the thin submuscular plane. The saline washed fat graft was placed into the pouch as a single piece. The incision line was repaired with 4/0 non-absorbable monofilament suture. Povidone-iodine dressing was applied.

*Group 2 (*n=12*):* Rats were placed in the prone position and restrained. The dorsal side of the cervical region was shaved so that it was caudal to both ears, which were determined to be mobile regions. A 1.5-cm incision was made in the coronal plane. A 1.5 × 1 cm pocket was created in the subcutaneous plane in the caudal direction. The harvested fat graft was washed with saline and placed in the pouch as a single piece. The incision line was repaired with 4/0 non-absorbable monofilament. A povidone-iodine dressing was applied.

### Sample Collection and Analysis

The study period was set at 6 weeks. During the study, 1 rat in Group 1 died on day 2 after surgery. After a 6-week waiting period, general anaesthesia was induced in all rats with 75 mg/kg ketamine and 10 mg/kg xylazine in the left intraperitoneal region. An incision was then made from the old scar line, and the transplanted fat grafts were dissected from the surrounding tissue. The rats were sacrificed after the procedure by a humane euthanasia method. For histopathological examination, the fat grafts were processed in 10% formaldehyde.

**Fig. 2:**
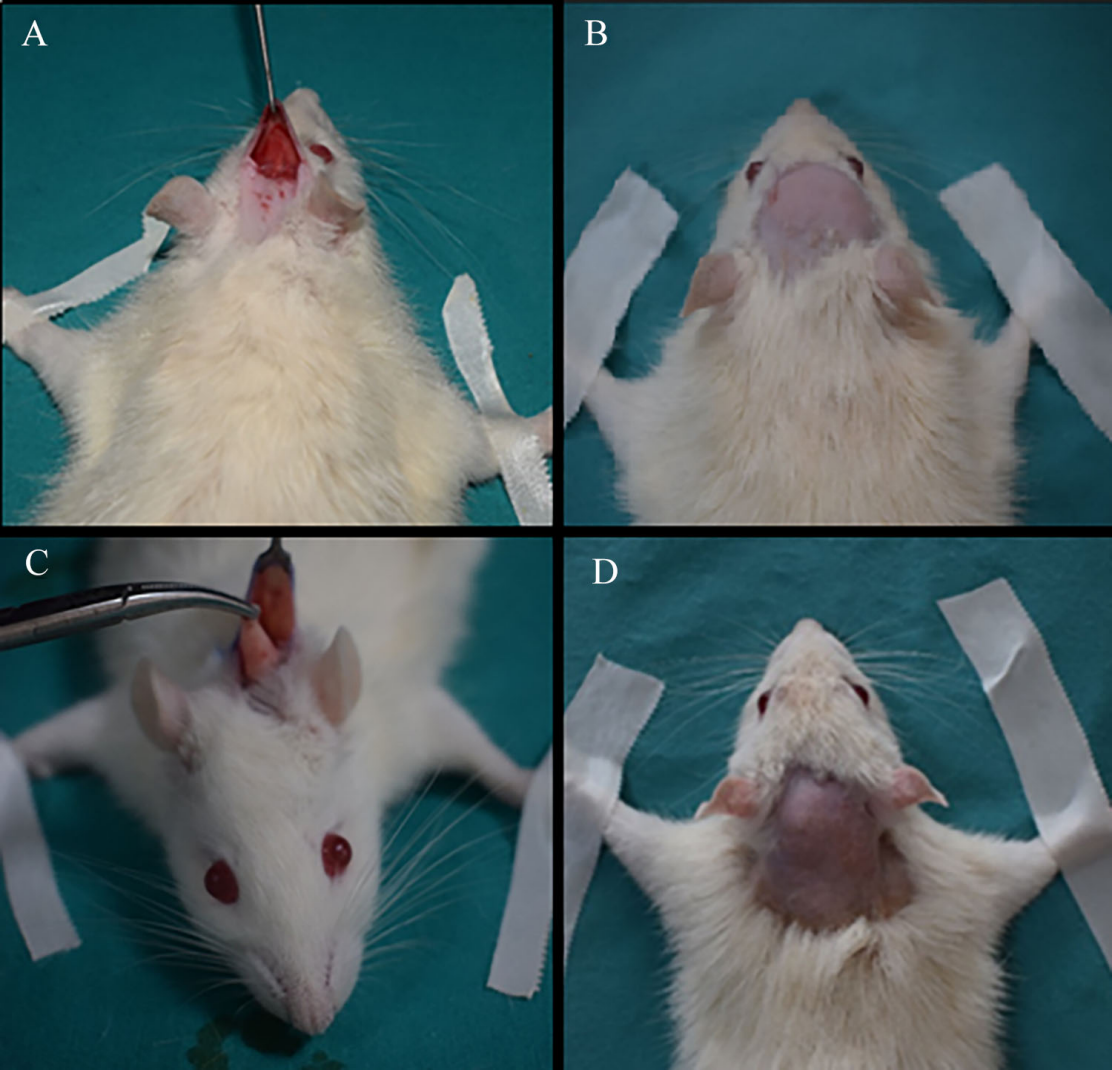
Fat graft transfer to immobile area in Group 1 rat (**A**), Group 1 recipient area at 6th week (**B**), fat graft transfer to mobile area in Group 2 rat (**C**), Group 2 recipient area at 6th week (**D**)

Photographs of the fat graft transplanted areas of Group 1 and Group 2 rats and the recipient area at the 6th week are shown in Fig. 2.

### Histopathological Examination

**Fig. 3:**
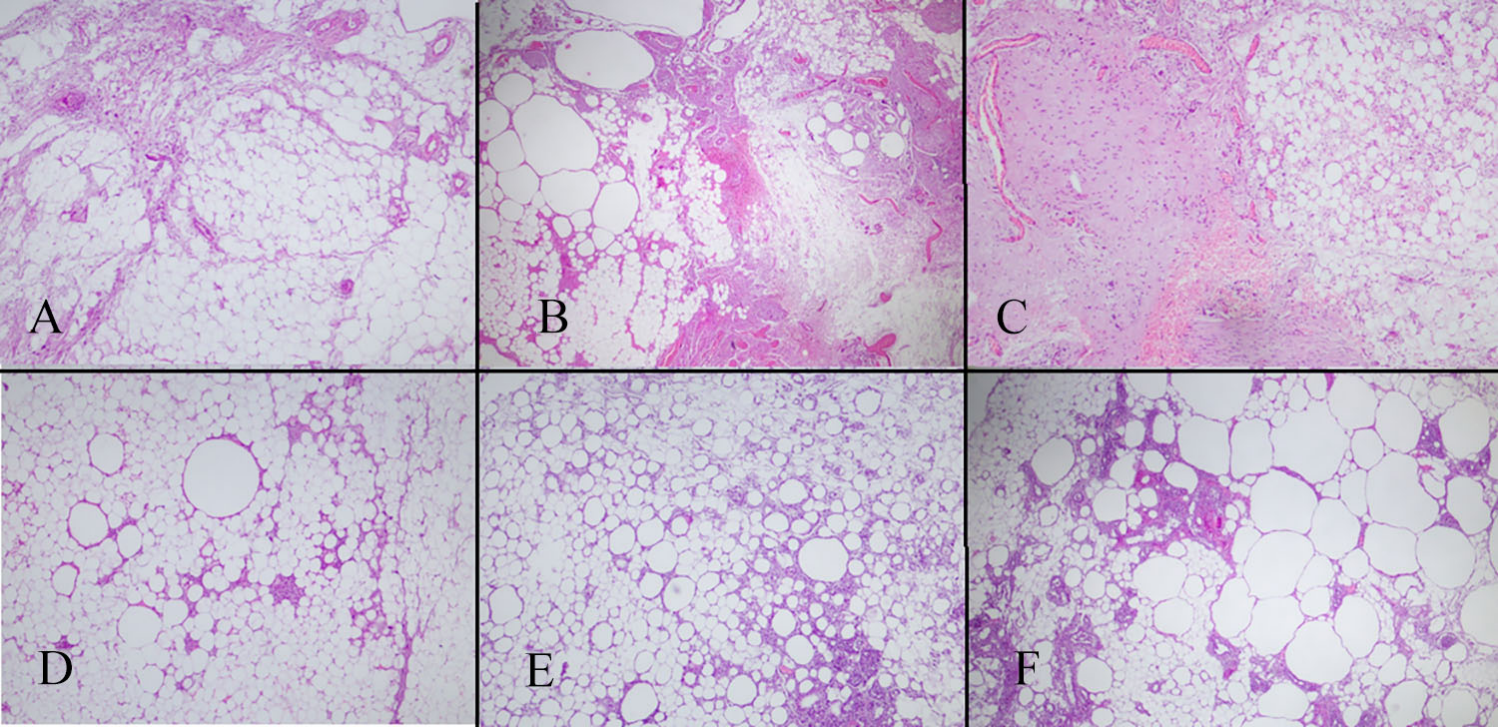
Cyst-vacuole formation and fibrosis (H&Ex400) (**A**: Score 1, **B**: Score 2, **C**: Score 3). Cyst-vacuole formation and inflammation (H&Ex400) (**D**: Score 1, **E**: Score 2, **F**: Score 3)

Histopathological examination was performed under a light microscope with haematoxylin and eosin staining by a single pathologist in a blinded manner. Five parameters were assessed in the histopathological evaluation. These parameters were normal adipose tissue, necrotic adipose tissue, inflammation, fibrosis, cyst-vacuole formation and vascular density. Semi-quantitative scoring was performed for vacuole/cyst formation, inflammation, fibrosis in these samples as 0: Absent, 1: Mild, 2: Moderate, 3: Severe (Fig. 3). The percentage of normal adipose tissue and the area of necrosis in the total tissue were determined. Vessel density was determined by counting luminal structures containing erythrocytes and/or endothelium in 10 consecutive high-power fields (× 400) and recording the highest number of vessels. Sections were photographed with a digital camera.

### Statistical Analysis

SPSS for Windows *v*.15.0 was used for statistical analysis. Median, minimum and maximum values of all outcomes were determined. Data analysis was performed using the Mann–Whitney *U* test, which is a non-parametric test used to compare independent variables. Values were compared between two groups. The correlation between numerical data was evaluated by Spearman’s correlation test. Results were considered significant when *P* < 0.05.

## Results

The numerical data of 11 rats in Group 1 and 12 rats in Group 2 could be included in the study. All numerical data obtained from our study are presented in Table 1. When the data in Group 1 were analysed among themselves using the Spearman correlation test, there was a positive correlation between the inflammation score and the necrosis ratio data (*p*:0.001), but no correlation was found between the other histopathological numerical data and the adipose tissue weight obtained from the rat. In Group 2, there was a positive correlation between the cyst-vacuole formation score and the necrosis ratio data (*p*:0.001) and between the inflammation score and the fibrosis score data (*p*:0.022). In Group 2, in contrast to the other group, there was a positive correlation between vascular density and the weight of adipose tissue obtained from the rats (*p*: 0.013).

**Table 1:** All numerical data obtained from our study

Groups	Normal adipose tissue ratio	Cyst-vacuole formation score	Inflammation score	Necrosis ratio	Fibrosis score	Vascular density	Adipose graft tissue at 6th week (g)
*Group 1*							
1	90	3	1	10	1	62	0,17
2	10	3	3	90	3	38	0,29
3	90	2	1	10	1	30	0,42
4	80	3	1	20	0	40	0,45
5	90	2	1	10	1	30	0,60
6	90	2	1	10	1	38	0,43
7	70	3	3	30	0	36	0,16
8	20	3	3	80	3	54	0,13
9	90	2	1	10	0	40	0,41
10	80	2	2	20	1	46	0,58
11	50	3	2	50	1	30	0,38
*Group 2*							
1	80	2	2	20	1	25	0,30
2	80	2	1	20	1	29	0,37
3	95	1	1	5	0	25	0,23
4	20	3	2	80	1	20	0,17
5	20	3	3	80	1	26	0,29
6	25	2	2	75	1	52	0,31
7	20	3	3	80	3	28	0,23
8	90	1	3	10	2	45	0,34
9	30	3	2	70	2	30	0,29
10	40	3	2	60	1	15	0,27
11	30	3	2	70	1	18	0,27
12	85	2	1	15	1	38	0,39

When the histopathological numerical data and adipose tissue weight obtained from rats were compared between Groups 1 and 2, vascular density was significantly higher in Group 1 (median: 38) than in Group 2 (median: 27) (*p*: 0.007), and adipose tissue weight obtained from rats was significantly higher in Group 1 (median: 0.42 g) than in Group 2 (median: 0.29 g) (*p*: 0.027). However, normal adipose tissue ratio (*p*:0.228), cyst-vacuole formation score (*p*:0.651), inflammation score (*p*:0.413), necrosis ratio (*p*:0.288) and fibrosis score (*p*:0.487) were not statistically different between these two groups.

## Discussion

Autologous fat grafting is widely used in plastic, aesthetic and reconstructive surgery. It is more advantageous than fillers due to its easy availability, non-allergenicity and low morbidity in the donor area [9]. The main disadvantage is that the resorption of fat grafts cannot be predicted [10]. Fat necrosis, fat cysts and calcifications are also disadvantages [9]. Therefore, many studies have been performed in the literature. However, there is no consensus on the ideal methods to increase the survival of fat grafts. A review of the studies in the literature aimed at increasing the viability of autologous fat grafts shows that different methods related to the harvesting, preparation and application of fat grafts have been investigated, as well as internal and external factors affecting fat grafts in the recipient site [11].

Reasons for reduced survival of fat grafts include early inflammation in the recipient site, oxidative stress and loss of adipocytes due to apoptosis in the late period [12]. Various agents have been used in fat graft applications to regulate local and systemic inflammation to increase the blood supply to the fat graft. Some of these agents are insulin, vitamin E, FGF, EGF, VEGF, erythropoietin and deferoxamine [13]. In order to increase the vascularity of the recipient site, there have been studies of the administration of growth factors, platelet-rich plasma, adipose-derived stem cells or preconditioning of the recipient site with various methods [14].

There are studies in the literature showing that the vascularity of the transplanted fat graft is derived from the recipient site [15]. It is known that exercise has a negative effect on the newly formed vessels in the recipient site. Some studies have shown that the differentiation and proliferation properties of ADSCs are negatively affected by the mechanical force in the moving area [16]. ADSCs have been reported to play a role in the survival of fat grafts [21].

It has been observed that experimental studies are more common than clinical studies in studies of fat graft viability. Comparison of study results between research groups is difficult due to the different applications used [22].

There are very few studies on the effect of movement of the recipient site on the fat graft. These studies have mostly used the denervation method. The denervation method restricts active movement, but various effects of nerve tracts are also involved [26, 28].

In the postoperative period, it can be predicted that the survival of the fat graft in the mobile area may be low. In clinical practice, for fat grafting to the face, it is recommended to restrict movement as much as possible in the early postoperative period [24, 25]. For fat grafting to the breast, it is recommended to reduce the use of the pectoralis major muscle [23].

In our study, we aimed to investigate the effect of the mobile and immobile area in the recipient site on fat graft survival in a new experimental model. The most commonly used recipient site in rat studies of fat graft survival is the pouch created in the subcutaneous region of the back and scalp [14]. In the rat studies reviewed, studies comparing fat graft recipient sites are rare. In many studies, the recipient site was found to be the same in the experimental groups [17, 18]. Studies in which fat grafts were placed in the cervical dorsal region of the rat were found in the literature, but no comparison with the immobilised area was observed. In one study, the recipient site was reported as the posterior cervical region, but the fat graft was placed in the interscapular area [19]. In our study, a new experimental model for fat graft recipient sites was defined. Two recipient sites were defined as mobile and immobile areas in the rat. These are adjacent areas. The blood supply to these two sites is provided by the same main vessel. In Group 1, the scalp area was chosen as the immobilised area, as it is often used in the literature. The frontal muscle is located on the bone in the scalp region chosen as the receptor region. The frontal muscle is the continuation of the galea aponeurotica and is known to be very weak in movement. This region is supplied by the anterior auricular artery, which is a branch of the external carotid artery. There are 7 cervical vertebrae in the cervical dorsal region selected as the mobile region. The range of motion of the cervical vertebrae is quite large. It is provided by the movement of the neck in different directions as the rat drinks water in the cage, self-cleans itself or feeds. As a result of these movements, there is a sliding movement between the neck muscles and the skin, which puts stress on the graft placed in the mobile area. It is known that an immobilised environment is important for increasing skin graft adhesion and vascularisation. Movement can damage newly formed vascular structures and reduce the viability of the transplanted graft [20]. The superficial muscles covering the cervical vertebrae are the levator auris longus and the platysma. These muscles are supplied by the posterior auricular artery, which is a branch of the external carotid artery. In our study, the scalp region and the cervical dorsal region, which were defined as the recipient area in our study, are supplied by the branches of the external carotid artery. Both recipient areas are supplied by branches of the same main artery. In Group 1, the incision in the scalp region, which was selected as the immobilised area, was made in the coronal plane anterior to both ears. After the incision, the pouch was formed anteriorly and the fat graft was placed in the subcutaneous pouch opened anterior to the incision. The incision line remained posterior to the fat graft. This minimised the contact of the fat graft with the incision scar, as blood circulation in the incision scar was reduced. In Group 2, the cervical dorsal region adjacent to the scalp region was chosen as the mobile region. The incision was made in the coronal plane behind both ears. The pouch was opened towards the caudal side of the incision. The fat graft was placed in the pouch opened in the subcutaneous plane over the cervical vertebrae of the rat. The fat graft was placed behind the incision scar to minimise contact with the incision scar. In the new experimental model, the relationship between fat graft survival and movement was evaluated. In Group 1, there was no similar sliding movement and no stress on the graft. This was reflected in our vascularisation results. The increase in vascularisation in Group 1 was statistically significant. However, there was no significant difference between the other criteria analysed. In rat and mouse studies, the waiting period after fat graft placement at the recipient site varies from 1 to 52 weeks [14]. In our study, a waiting period of 6 weeks was preferred. Statistical analysis of the data showed no significant result for weight. There was no significant difference in the rates of cyst-vacuole formation, fibrosis, inflammation, normal fat and necrosis between the two groups. In our study, a significant increase in vascular density was found in the fat graft given to the immobile area compared to the fat graft given to the mobile area. Many studies in the literature have aimed to increase the vascularity of the fat graft. Long-term results have shown an increase in fat graft survival with increased vascularity. At the same time, our study defined a new experimental rat model. Our study has shown that mobile regions affect the survival outcome of fat graft applications. We believe that our new experimental model can guide new fat grafting studies in the literature. Studies to increase vascularity can be used in our new experimental model.

## Conclusion

In our study evaluated the relationship between fat graft survival and movement, using a new experimental rat model. We found that placing the fat graft in an immobile area resulted in a statistically significant increase in vascularization compared to the mobile area. It was also found that the weight of the fat graft at the end of week 6 was statistically higher in Group 1. However, there were no significant differences observed in other criteria analysed, such as weight, cyst-vacuole formation, fibrosis, inflammation, normal fat and necrosis. Overall, our findings suggest that increasing vascularity can improve the long-term survival of fat grafts. We believe that our novel experimental model can serve as a valuable guide for future studies focusing on enhancing vascularity in fat grafting procedures.
